# Proteolytic ENaC activation in health and disease—a complicated puzzle

**DOI:** 10.1007/s00424-021-02644-w

**Published:** 2021-11-20

**Authors:** Mike Althaus, Rene Yufenyuy Lawong

**Affiliations:** Institute for Functional Gene Analytics, Department of Natural Sciences, Bonn-Rhein-Sieg University of Applied Sciences, Von-Liebig-Strasse 20, 53359 Rheinbach, Germany

The epithelial sodium channel (ENaC) is a heterotrimeric ion channel that plays a key role in sodium and water homeostasis in tetrapod vertebrates. In the aldosterone-sensitive distal nephron, hormonally controlled ENaC expression matches dietary sodium intake to its excretion. Furthermore, ENaC mediates sodium absorption across the epithelia of the colon, sweat ducts, reproductive tract, and lung. ENaC is a constitutively active ion channel and its expression, membrane abundance, and open probability (P_O_) are controlled by multiple intracellular and extracellular mediators and mechanisms [9]. Aberrant ENaC regulation is associated with severe human diseases, including hypertension, cystic fibrosis, pulmonary edema, pseudohypoaldosteronism type 1, and nephrotic syndrome [9].

**Fig. 1:**
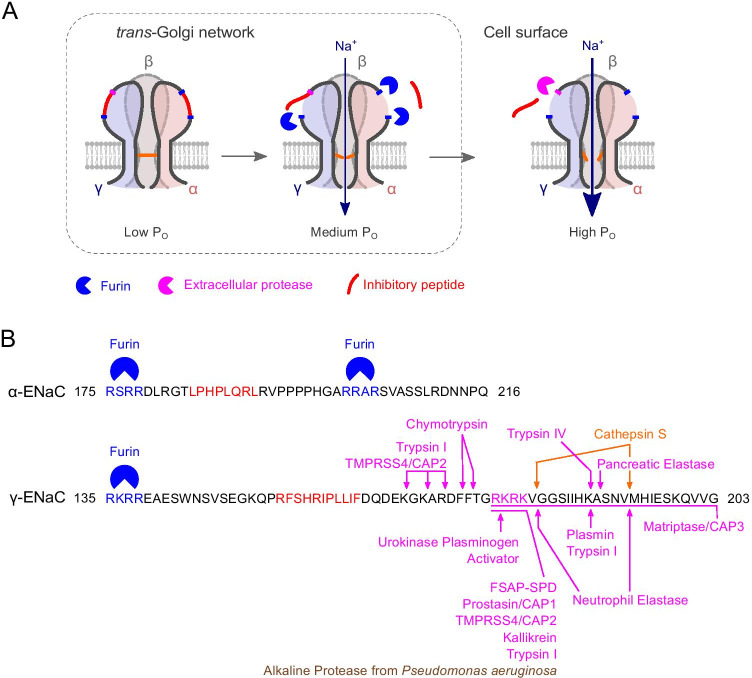
ENaC activation by intra- and extracellular proteases. **A** Cartoon illustration of ENaC cleavage by furin within the *trans*-Golgi network and extracellular proteases at the cell surface. Please note that αβγ-ENaC assembles in a counter-clockwise subunit orientation [7]. P_O_, open probability. **B** Peptide sequences of the α- and γ-subunits of human ENaC showing the regions containing the inhibitory peptides within the extracellular loop. Furin consensus sites are shown in blue; the inhibitory peptides [7] are shown in red. The figure shows extracellular proteases cleaving the γ-subunit whose cleavage sites have been identified by mutagenesis studies [4–6]: Serine proteases are shown in magenta, cysteine proteases in orange, and metalloproteases in brown. CAP, channel-activating protease (alternative name for the indicated proteases); FSAP-SPD, factor VII activating protease—serine protease domain

Canonical ENaC assembles in the αβγ-subunit combination. Its unique characteristic is the link between P_O_ and cleavage of ENaC subunits by proteases [5] (Fig. 1a). The α- and γ-subunits contain inhibitory peptides embedded in their extracellular domains, which lock ENaC in a low-P_O_ state. The α-subunit inhibitory peptide is flanked by cleavage sites for the endoprotease furin. Furin is present in the *trans*-Golgi network and cleaves the α-subunit twice, removing its inhibitory tract. The γ-subunit contains one furin cleavage site; therefore, the inhibitory tract remains attached to the γ-subunit until ENaC reaches the plasma membrane. Furin-processed ENaCs have an intermediate P_O_. The second cut releasing the γ-subunit inhibitory peptide is mediated by extracellular proteases (Fig. 1). Fully cleaved ENaCs display a high P_O_. The study by Artunc and colleagues adds factor VII activating protease (FSAP) to the list of ENaC-activating serine proteases [1]. Using *Xenopus* oocytes expressing human αβγ-ENaC, the authors demonstrate that a recombinant serine protease domain (SPD) of FSAP potently activates ENaC in a concentration- and time-dependent manner, whereas a metabolically inactive FSAP-SPD does not. Mutating a polybasic RKRK motif C-terminal to the ENaC γ-subunit inhibitory tract abolishes ENaC activation by FSAP-SPD.

Is proteolytic control of ENaC P_O_ important in health and disease? In lung epithelia, the balance between membrane-bound proteases and soluble protease inhibitors has been suggested to adjust ENaC P_O_ and, consequently, transepithelial sodium and water absorption, to the volume of liquid lining the airways and alveoli. Excess protease in the airway surface liquid contributes to airway dehydration in cystic fibrosis [10], whereas impaired ENaC cleavage promotes edema formation [8]. Physiological ENaC control by proteases in the kidney is less clear. While ENaC subunits are cleaved in human kidneys [11], it remains unclear whether ENaC cleavage is a specific regulatory mechanism adjusting channel P_O_ to sodium re-absorption. The situation changes when proteases accidentally encounter ENaC. During acute nephrotic syndrome (NS), serine proteases or their zymogenic forms (e.g., plasminogen) are filtered into pre-urine. Active proteases can cleave ENaC in the distal nephron, thereby enhancing ENaC P_O_, resulting in sodium retention and edema, both hallmarks of NS. The identity of ENaC-activating proteases is not completely understood and the contribution of urinary plasmin to sodium retention is under debate [3]. The study by Artunc and colleagues demonstrates that active FSAP is present in urine of patients with NS and in mice with doxorubicin-induced NS [1]. While serine protease inhibitors protect mice from sodium retention in this model [2], FSAP-deficient mice do not show altered sodium retention or ENaC cleavage in comparison with wild-type mice [1]. These experiments suggest that FSAP alone does not cause ENaC-mediated sodium retention in this mouse model and imply a mechanism compensating for FSAP deficiency or an ENaC-activating protease cocktail in nephrotic urine. Identification of the main proteases causing ENaC activation in NS is an important task that might pave the way for the development of specific therapeutic strategies. The study by Artunc and colleagues provides an experimental blueprint aiming to complete this puzzle.
